# Linguistic Analysis of Generic-Generic Drug Name Pairs Prone to Wrong-Drug Errors for which Tall-Man Lettering is Recommended

**DOI:** 10.1007/s43441-023-00526-0

**Published:** 2023-05-12

**Authors:** Gail B. Karet

**Affiliations:** Senior Scientist, American Medical Association, United States Adopted Names Program, Suite 39300, 330 N. Wabash Ave, Chicago, IL 60611-5885 USA

**Keywords:** Computational linguistics, Wrong-drug errors, Medication errors, Generic names, Tall-man lettering, Mixed case lettering

## Abstract

**Objective:**

The Institute for Safe Medication Practices (ISMP) and the United States Food and Drug Administration (FDA) disseminated widely used lists of drug name pairs involved in wrong-drug errors, for which they recommended tall-man lettering (TML). Linguistic similarity is believed responsible for confusion of these drugs. This study aims to quantify linguistic similarity and other linguistic properties of these generic-generic name pairs.

**Methods:**

The FDA’s Phonetic and Orthographic Computer Analysis (POCA) software was used to generate numerical similarity scores for the generic-generic name pairs on these lists and to identify conflicts between these names and the names of other marketed products. Within each pair, differences in name length and the number of identical prefix (initial) letters and suffix (final) letters were determined.

**Results:**

The selected pairs shared a mean of 2.5 (± 1.8) identical prefix letters and 3.2 (± 2.9) identical suffix letters. The mean POCA score 69.5 (± 9.7), indicated moderate-to-high similarity. POCA scores for individual pairs ranged from 90 (most similar) to 46 (least similar). Individual names averaged 11.2 (± 9.1) high-similarity conflicts with names of other marketed drugs.

**Conclusions:**

POCA analysis could be a valuable tool in determining whether linguistic similarity contributes to specific wrong-drug errors. The finding of 11.2 (± 9.1) high-similarity conflicts with names of other marketed drugs is more than for candidate names USAN accepts and suggests the names on the FDA and ISMP lists are linguistically problematic.

## Introduction

Medical errors are a long-standing and important problem in patient care [1] affecting as many as 1 in 20 patients [2]. The cost of medical errors, including lost income and productivity, disability, and additional care, was estimated in 2015 to be $42 billion worldwide [3]. Errors involving medications or drugs account for the largest share (25%) of preventable errors [2]. Wrong-drug errors, when patients are prescribed one drug and receive another, may cause injury, hospitalization, and death.

Similarity of drug names, packaging, uses, labels, and other factors may increase the likelihood of wrong-drug errors. Many concerns have been raised about drug names that look or sound too much alike [4–6]. When name similarity is believed to cause a wrong-drug error, it is called a look-alike, sound-alike (LASA) drug name error. Although databases of medication errors and adverse events exist, [7] published reports describing wrong-drug errors are often anecdotal and not quantitative [4]. The United States Food and Drug Administration (FDA) collects over 100,000 reports of suspected medication errors annually through its MedWatch Program [8]. In addition, the Institute for Safe Medication Practices (ISMP) collects, investigates, and disseminates anecdotal reports about specific wrong-drug errors and other medication errors.

Starting as early as the 1970’s, there have been attempts to disseminate lists of drug names that might look or sound alike [9]. In 2001, the FDA began its Name Differentiation Project to evaluate post-marketing reports of name pair confusion. This resulted in the *FDA List of Established Names Recommended to Use Tall-Man Lettering (TML)* [10]. In tall-man lettering, which is also called mixed case lettering, portions of drug names are capitalized (e.g., glipiZIDE vs. gliBURIDE) with the expectation that this will call attention to the potential for medication errors and aid in differentiating a drug name from another name. The ISMP compiled another list of medications associated with LASA errors, recommending specific tall-man lettering for additional drug name pairs, and continues to update this list based on interviews with healthcare providers and when drugs become unavailable in the US [11].

Tall-man lettering has gained wide acceptance [12]. The expectation is that capitalizing the parts of a name pair that are different will visually differentiate similar drug names. Despite its use, there is not strong evidence that it prevents medication errors. Studies have examined the effectiveness of tall-man lettering, as well as other interventions such as bold-face type, in preventing medication errors [13–16]. A recent, systematic review of interventions to prevent LASA errors found that tall-man lettering was marginally effective. The authors suggested that its efficacy was the result of a “quasi-placebo effect,” with users deriving more benefits when they were aware of its use and purpose [14].

United States Adopted Names (USAN) designations are chosen after multi-party negotiations requiring consensus between the USAN Council, the submitting firm and the WHO’s International Nonproprietary Names (INN) Programme [17]. At USAN, the goal is to develop drug names that fit the nomenclature scheme, are pronounceable, are free of linguistic problems in major world languages, and do not conflict with trademarks. That said, the safety of names—and the lowest possible risk of LASA errors—is USAN’s most important goal.

For many years, determining linguistic similarity depended entirely on visual inspection methods. USAN still uses these methods in screening proposed names. USAN judges two drug names to be potentially similar when they share the same suffix, or USAN stem, and at least two of the letters at the beginning of a drug name are shared.

USAN began using the FDA’s Phonetic and Orthographic Computer Analysis (POCA) software [18, 19] to screen drug names in 2018, and this tool quantifies the degree of similarity of two drug names. POCA is based on an A-Line computational method [19, 20]. POCA provides similarity scores, ranging from a low of 0 (no similarity) to a high score of 100 (an exact match), when spoken (phonetic) or written (orthographic) [18, 19]. The combined score is an average of the phonetic and orthographic scores. As of 2021, FDA documentation, which is based on analysis of proprietary (trade) names, classified two names with a combined score ≥ 70 as highly similar, a score ≥ 55 and < 70 as moderately similar, and scores < 55 as indicating low similarity [19]. In screening USAN candidates, POCA scores ≥ 70 warrant consideration of a conflict and scores ≥ 80 are typically disqualifying. In 2021, the FDA released an online version of its POCA tool, making it readily available, and free, for anyone to use [19].

Computational linguistic software has been used to assess generic names for a risk of medication errors, [5, 21] but published studies have not employed POCA analysis to investigate linguistic similarity of the generic-generic name pairs on the FDA and ISMP lists. POCA analysis was conducted on the trade name pairs on these lists [22]. Because quantitative information about similarity is potentially useful in directing medication error prevention efforts, and because these names are known to have been involved in wrong-drug errors, the POCA tool was applied to the generic names on the lists disseminated by the FDA and the ISMP.

The objective of this work was to quantify the degree of linguistic similarity for the generic-generic name pairs for which the FDA and ISMP recommended tall-man lettering. It is hoped that quantitative information about name similarity will be useful to those trying to prevent wrong-drug errors, improving measures to prevent wrong-drug errors involving these drugs.

## Methods

### Selection of Drug Name Pairs

Names were selected from the ISMP and FDA lists of name pairs for which tall-man lettering was recommended, as of November, 2020 [10, 11]. The name pairs on the lists include generic-generic pairs, generic-proprietary pairs, and proprietary-proprietary pairs. Only generic-generic name pairs were selected for this analysis. USAN selected 43 names on the list disseminated by the FDA’s Name Differentiation Project and 117 names on the ISMP list for review. With some names appearing in more than one pair and some on both lists, 142 individual names were evaluated in total. There were 95 generic-generic name pairs, 21 on the FDA’s list and 74 on the ISMP’s list.

### Age of Drug Names

A search of the 2021 online version of the USP Dictionary of USAN and International Drug Names [23] was conducted to determine the date of adoption of each drug name. The oldest names were USP and/or National Formulary designations predating the 1962 inception of the USAN Program. In this case, the exact date was not established but is known to be before 1962.

### Determining the USAN stem

To determine the USAN stem (a suffix that suggests the action or use of a drug), each name was compared to the USAN stem list [24]. A name was judged to contain a specific USAN stem when it incorporated the same string of letters, in the same position in the name where the stem normally appears.

### Length of Drug Names

The number of letters in each name was counted, with the results double-checked and tracked in an Excel spreadsheet. The difference in the name length was calculated for each pair by subtracting the length of one name from the length of the other and taking the absolute value.

### Number of Shared Letters

The two names in each name pair were compared side-by-side, starting from the first letter, and working backwards from the last letter. The number of identical prefix letters and suffix letters were counted.

### POCA Analysis of Name Pairs

Version 4.3 [25] of the FDA’s Phonetic and Orthographic Computer Analysis (POCA) was used to perform computational linguistic analysis. This software was set up on an internal server by the American Medical Association’s (AMA’s) IT department. The Single Name Direct Search tool, which calculates a comparison score for two names, was used to determine orthographic, phonetic, and overall scores for each name pair.

### POCA Analysis of Individual Names

Each name was entered individually into the Drug Name Search Tool of Version 4.3 of POCA [25]. This tool searched the names in the Drugs@FDA and RxNorm databases, [26, 27]. POCA provides a list of conflicts with names in the databases, ordered from most to least similar by their combined POCA score, and tabulates the number of highly-similar and moderately similar name conflicts.

### Statistical Analysis

The functions in an Excel spreadsheet were used to calculate mean (AVERAGE), median (MEDIAN) and mode (MODE) for groups of data. Standard deviations were also calculated in Excel, using the STDEV.P function.

## Results

### Adoption Dates

**Figure 1:**
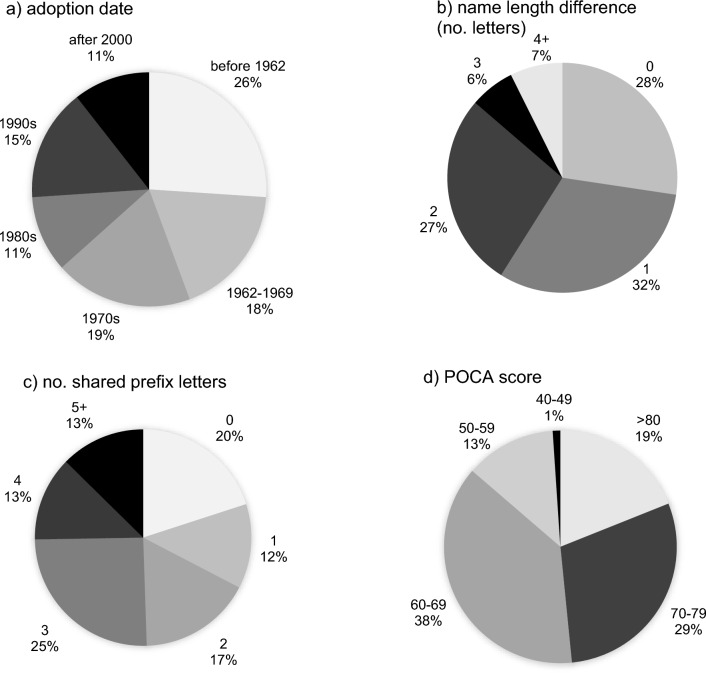
Distribution of key characteristics for name pairs: **a** adoption/inception date **b** difference in the length of the names (number of letters) **c** number of shared prefix letters and **d** combined POCA score.

A search of the USP Dictionary for each name’s adoption date found that most drug names for which FDA and ISMP recommended tall-man lettering were selected several decades ago. A little over one-quarter of the names, 26%, (Fig. 1a) predate the USAN Program’s inception in 1962. Approximately two-thirds of the names (63%) predated the USAN Program or were adopted as USAN between 1962 and 1979. Only 11% were adopted as USAN in 2000 or later, and only 2 were adopted as USAN after 2010 (The latest was 2012.). All were adopted before 2018, when the USAN Program began using POCA tools.

### Name Length and Shared Prefixes and Suffixes

The names on the FDA and ISMP lists were, on average, 11.0 (± 2.4) letters long (Table 1). Names on the FDA list were, on average, slightly longer, 12.3 (± 2.9) letters, than those on the ISMP list, 10.7 (± 2.1) letters.

**Table 1:** Mean POCA scores and the results of visual inspection, with standard deviations in parentheses, for the name pairs on the FDA and ISMP lists

	All name pairs	FDA list only	ISMP list only
Combined POCA score	69.5 (± 9.7)	72.1 (± 9.3)	68.6 (± 9.7)
Orthographic score	75.1 (± 9.6)	77.6 (± 8.3)	74.4 (± 9.8)
Phonetic score	63.9 (± 13.2)	66.6 (± 12.9)	62.9 (± 13.3)
Length of names (no. letters)	11.0 (± 2.4)	12.3 (± 2.9)	10.7 (± 2.1)
Name length difference (no. letters)	1.4 (± 1.5)	0.9 (± 0.9)	1.5 (± 1.5)
No. identical letters in prefix	2.5 (± 1.8)	3.6 (± 2.0)	2.1 (± 1.7)
No. identical letters in suffix	3.2 (± 2.9)	3.4 (± 2.1)	3.1 (± 3.0)

The lengths of the names in each pair were similar. The mean difference in the length of the names within the pairs was 1.4 (± 1.5) letters. About one-quarter of the name pairs (27%) were the same length (Fig. 1b).

A mean of 2.5 (± 1.8) prefix letters and 3.2 (± 2.9) suffix letters were identical within the name pairs. Roughly half the name pairs began with a consecutive string of 3 or more identical prefix letters (Fig. 1c). The FDA names had 3.6 (± 2.0) identical prefix letters, as opposed to the ISMP names, with 2.1 (± 1.7 prefix letters).

Just under one-third of the name pairs (31%) shared a USAN stem, fixed syllables that indicate use or action. A shared stem increases the number of shared letters, usually in the suffix, and indicates similar uses or mechanisms of action. The remainder did not have a USAN stem or had different stems.

### POCA Analysis of Name Pairs

The mean combined score for all name pairs was 69.5(± 9.7) (Table 1). This mean is near the threshold for a high-similarity conflict, ≥ 70 [18]. The mean orthographic score, 75.1(± 9.6) was in the high-similarity range and higher than the mean phonetic score, 63.9(± 13.2). The mean combined score for name pairs on the FDA list was slightly higher, 72.1(± 9.3), than for the ISMP list, 68.6(± 9.7).

The vast majority of the generic-generic name pairs, 94%, had a POCA score ≥ 55. Thus, nearly all of them scored as moderately or highly similar. As shown in Fig. 1d, 18(19%) had a POCA score of 80 or higher. Almost half, 46(48%), scored ≥ 70. There were 13 name pairs (14%) scoring ≤ 60 and 35(36%) scoring ≤ 65.

Pairs with the highest and lowest combined scores are shown in Table 2. The HYDROXYprogesterone and medroxyPROGESTERone pair had the highest score (90). Some other name pairs with very high POCA scores were PENTobarbital and PHENobarbital (89), cycloSERINE and cycloSPORINE (88), prednisoLONE and predniSONE (85), and sAXagliptin and SITagliptin (85). The metFORMIN and metroNIDAZOLE (46) pair had the lowest score.

**Table 2:** Name pairs with the highest and lowest POCA scores

Most similar pairs		POCA score	Least similar pairs		POCA score
HYDROXYprogesterone	medroxyPROGESTERone	90	medroxyPROGESTERone	methylPREDNISolone	58
PENTobarbital	PHENobarbital	89	cefOXitin	cefTAZidime	58
cycloSERINE	cycloSPORINE	88	hydroCHLOROthiazide	hydrOXYzine	58
prednisoLONE	predniSONE	85	traMADol	traZODone	58
sAXagliptin	SITagliptin	85	cefoTEtan	cefTAZidime	56
chlorproMAZINE	chlorproPAMIDE	84	hydrALAZINE	hydroCHLOROthiazide	56
sulfADIAZINE	sulfaSALAzine	84	HYDROmorphone	morphine	56
valACYclovir	valGANciclovir	84	cefTRIAXone	ceFAZolin	54
ARIPiprazole	RABEprazole	82	OLANZapine	QUEtiapine	54
DULoxetine	FLUoxetine	82	risperiDONE	rOPINIRole	53
ISOtretinoin	tretinoin	82	ALPRAZolam	clonazePAM	50
metyraPONE	metyroSINE	82	mitoMYcin	mitoXANTRONE	50
NIFEdipine	niMODipine	82	metFORMIN	metroNIDAZOLE	46
raNITIdine	riMANTAdine	82			
DAUNOrubicin	DOXOrubicin	81			
DACTINomycin	DAPTOmycin	81			
dimenhyDRINATE	diphenhydrAMINE	80			
vinBLAStine	vinCRIStine	80			

### POCA Analysis of Individual Names

POCA’s Drug Name Search Tool found the individual names had a mean of 11.2 (± 9.1) high-similarity conflicts (scores ≥ 70). The drugs with the most high-similarity conflicts were dopamine (52), mitomycin (37), tizanidine (36), dobutamine (35), clonidine (33), penicillin (33), ranitidine (33) and penicillamine (30). Almost half the names (46%) had 10 or more conflicts scoring ≥ 70, and 30% had 15 or more. The majority (61%) had at least one conflict with a POCA score ≥ 80.

Many of the name conflicts that POCA’s Drug Name Search Tool found had a score higher than the conflict within the name pair. While 28% of name-pair conflicts were the highest-scoring conflict, for 72% of the names the Drug Name Search Tool found one or more conflicts with higher POCA scores.

## Discussion

Visually screening names is still important and useful, and names look more similar if they are similar in length and begin and end with similar, or the same, letters [5, 6]. Many name pairs analyzed shared several prefix and suffix letters and were nearly or exactly the same length.

Complaints about the length and complexity of drug names are common [28, 29]. The names for which FDA and ISMP recommended tall-man-lettering were comparable to or only slightly longer than the mean for a random sample of INNs, 11.0 (± 2.4) letters versus 10.54 (± 1.73) letters, respectively [30]. Thus, unusually long length does not lead to LASA errors for this group of drug names. Whether or not longer names lead to medication errors, USAN still strives for shorter names for other reasons, such as ease of pronunciation, when assigning them is possible within the constraints of the nomenclature system.

Most name pairs had one or more characteristics associated with high similarity: POCA scores ≥ 70, identical length, or the same prefix letters. However, a substantial minority had lower POCA scores, different name lengths and/or fewer shared prefix and suffix letters, characteristics consistent with moderate or low similarity. USAN often views moderate similarity conflicts as not problematic when screening candidate names, but the seriousness of a conflict depends on the circumstances. In screening name candidates, USAN staff routinely finds multiple conflicts with names in the RxNorm and Drugs@FDA databases scoring ≤ 65, and they are so common that they do not disqualify a candidate name. Because there are more than 10,000 generic names [23], and because members of the same drug class must share the same USAN stem or suffix, almost every name candidate has multiple conflicts with other names scoring ≤ 65.

The most and least similar name pairs (Table 2), as determined by POCA analysis, may require different approaches to prevent wrong-drug errors. Those with very high similarity scores are the most likely to be involved in wrong-drug errors because of name similarity. A low POCA score indicates that two drug names are not highly similar, and it may be useful to conduct additional studies to look for causes of wrong-drug errors other than name similarity.

POCA analysis also provides information that might be useful in guiding error-prevention strategies for specific pairs, such as morphine and HYDROmorphone. These two drugs continue to be involved in wrong-drug errors, causing harm including death to patients, despite measures to differentiate these two names [31–34]. The combined POCA score for morphine and HYDROmorphone (56) is on the low end of the moderate similarity range and well below the threshold that USAN considers to be a problematic conflict in candidate names. This raises the possibility that factors other than name similarity may account for the continued confusion of morphine and HYDROmorphone. However, the orthographic score for the morphine and HYDROmorphone pair (76) is close to the average for the generic-generic name pairs included in this study and much higher than the phonetic score (36). Consequently, if name similarity is contributing to the confusion of these two drugs, efforts to differentiate morphine and HYDROmorphone when written might be more useful than efforts to differentiate them phonetically.

Individual names on the ISMP and FDA lists may be linguistically problematic, even when the score for a name pair does not indicate high similarity. The names on the ISMP and FDA lists had a very high number of high-similarity conflicts with names in the RxNorm or Drugs@FDA databases. For example, over half the names had at least one conflict with a POCA score ≥ 80, indicating a highly problematic conflict. Conflicts with names in the RxNorm and Drugs@FDA databases scoring ≥ 80 are uncommon in POCA screens of USAN candidates, but they were common for the names for which ISMP and FDA recommended tall-man lettering.

The number of conflicts scoring ≥ 70 but < 80 that USAN accepts in a candidate name varies according to the stem and other nomenclature considerations. For example, one or two conflicts scoring ≥ 70 but < 80 are acceptable in most cases, and several may be acceptable for names assigned to crowded stem classes (e.g., -tinib) with dozens of members. The USAN Council, however, typically rejects candidate names with as many conflicts scoring ≥ 70 as the average number found for the drug names in this study, 11.2 (± 9.1).

A study of the proprietary name pairs on the FDA and ISMP lists found that 75% of the pairs had POCA scores ≥ 50, more than half had shared strings of ≥ 3 prefix letters, and that the pairs were the same or a similar number of letters in length [22]. Most trade name pairs were in the moderate similarity category, and one-quarter had scores indicating low similarity. Generic-generic name pairs may be slightly more similar than proprietary name pairs, but in generic naming there are additional constraints, such as the need to use the same suffix for members of the same drug class or restrictions against specific letters (*e.g.*, h, k). These restrictions may affect the linguistic properties of generic name pairs [30].

Without quantitative linguistic analysis, it has been the experience of USAN Program staff that whether two drug names “look similar” or “sound similar” is subjective and depends on the languages spoken by those making the judgement, among other factors. Consequently, while qualitative judgements are a useful starting point, quantitative methods may be valuable in understanding whether wrong-drug errors are caused by name similarity.

Because POCA was developed to screen proprietary names before regulatory approval, further work is needed to validate it as a tool for identifying problematic name pairs in clinical practice, or for targeting error-prevention measures. However, this additional work might be particularly valuable now that a free, online version of POCA is available.

## Summary

Quantitative tools, such as comparing the number of shared letters, name length, and POCA analysis, are valuable for judging the similarity of two generic drug names. When applied to widely disseminated lists of name pairs for which tall-man lettering is recommended to reduce wrong-drug errors, POCA analysis found about half the name pairs were highly similar, with the remainder having mainly moderate similarity. Many of the names on these lists were problematic, having numerous conflicts with other names of marketed drugs. This suggests that the relationship between drug name similarity and wrong-drug errors is more complex than is often assumed. More study is needed to understand how the linguistic properties of drug names affect medication errors.
